# Physical ACtivity facilitation for Elders (PACE): study protocol for a randomised controlled trial

**DOI:** 10.1186/s13063-015-0610-8

**Published:** 2015-03-13

**Authors:** Gemma S Morgan, Anne M Haase, Rona Campbell, Yoav Ben-Shlomo

**Affiliations:** School of Social and Community Medicine, 39 Whatley Road, Bristol, BS8 2PS UK; School of Policy Studies, 8 Priory Road, Bristol, BS8 1TZ UK

**Keywords:** Physical activity, Ageing, Disability, Randomised controlled trial, Complex intervention, Self-determination theory, Physical performance

## Abstract

**Background:**

As people live longer, their risk of disability increases. Disability affects quality of life and increases health and social care costs. Preventing or delaying disability is therefore an important objective, and identifying an effective intervention could improve the lives of many older people. Observational and interventional evidence suggests that physical activity may reduce the risk of age-related disability, as assessed by physical performance measures. However it is unclear what approach is the most cost-effective intervention in changing long-term physical activity behaviour in older adults. A new theory-driven behavioural intervention has been developed, with the aim of increasing physical activity in the everyday lives of older adults at risk of disability. This pilot study tests the feasibility and acceptability of delivering this intervention to older adults.

**Methods/Design:**

A randomised controlled trial (RCT) design will be used in the pilot study. Sixty patients aged 65 years and older will be recruited from primary care practices. Patients will be eligible to participate if they are inactive, not disabled at baseline, are at risk of developing disability in the future (Short Physical Performance Battery score <10/12), and have no contraindications to physical activity. Following baseline measures, participants will be randomised in a 2:1 ratio to the intervention or to a control arm and all participants will be followed-up after 6 months. Those randomised to the intervention arm will receive sessions with a trained Physical Activity Facilitator, delivering an intervention based on self-determination theory. Control participants receive a booklet on healthy ageing. The main outcomes of interest are recruitment, adherence, retention and acceptability. Data will also be collected on: self-report and accelerometer-recorded physical activity; physical performance; depression; wellbeing; cognitive function; social support; quality of life, healthcare use, and attitudes to physical activity. A mixed-methods process evaluation will run alongside the RCT.

**Discussion:**

The intervention, if effective, has the potential to reduce disability and improve quality of life in older adults. Before proceeding to a full-scale trial a pilot trial is necessary to ensure intervention feasibility and acceptability, and that the intervention shows evidence of promise.

**Trial registration:**

Current Controlled Trials ISRCTN80470273. Registered 25 October 2013.

## Background

### Ageing, disability, and physical activity

Life expectancy in the UK has been increasing, and recent advances in medical science have reduced deaths from heart disease and cancer, resulting in a greater population of older adults with increasing prevalence of comorbidities. Around one third of babies born in 2013 are expected to reach their 100th birthday [1], and in 2011 adults aged 65 years and over comprised 16% of the population [2]. It is important that efforts to extend life do not overshadow efforts to enable older adults to live independently and to function well into old age. Failing to achieve this has consequences for both the individual suffering from a diminished quality of life, and for a society trying to contain the costs associated with an increased demand for health and social care [3].

Successful ageing necessitates the prevention of physical disability. The classic model of disability by Nagi [4] described a step-wise pathway from physiological pathology to performance limitations, which affect social roles and tasks, causing disability. Verbrugge and Jette [5] and more recently Rejeski *et al*. [6] have identified key modifiers along the disability pathway, including lifestyle behaviours and psychosocial attitudes. Researchers have been interested in identifying those key modifiers or risk factors, as they may provide an opportunity to intervene and prevent, or at least delay, age-related disability.

There is now a considerable body of observational research suggesting that physical activity is associated with physical function in later life, and that physical activity interventions in community-dwelling older adults may prevent or delay disability. Recommendations from the 2011 Chief Medical Officers’ report on physical activity advised that adults over 65 years undertake at least 2.5 hours of moderate intensity physical activity per week, including sustained bouts of 10 minutes [7], yet we know from cross-sectional surveys that many older adults are not meeting this target [8]. It therefore remains a key challenge to identify a cost-effective intervention that can increase physical activity in the long-term and can prevent disability in older adults in the UK.

### Physical Activity Facilitation (PAF)

An existing intervention, successfully used to increase physical activity in adults with depression, has been modified and developed for application to a population of older adults at risk of disability. Physical Activity Facilitation (PAF) was first developed for the TREAD trial, aiming to help adults with depression by using PAF facilitators to deliver behaviour change techniques using motivational interviewing and counselling strategies [9]. In the TREAD study patients randomised to the intervention (PAF) arm were twice as likely to be physically active compared to controls at 8 months, and this effect was sustained beyond cessation of the intervention. The PAF intervention is based upon self-determination theory [10], which purports that for an individual to modify their behaviour three core psychological needs must be met: the need for autonomy (having control and choice over activity); for competence (feeling capable about doing something); and for relatedness (feeling connected to and supported by others). The rationale and design of the original PAF intervention is described elsewhere [11]. In summary, the PAF facilitator acts as the main agent of change, and aims to address the core psychological needs of participants through face-to-face and telephone sessions.

Older adults at risk of disability may share common features with adults with depression such as loneliness, lack of confidence, and apathy and it is, therefore, possible that the PAF intervention will be effective in changing behaviour in the older adult population. The original PAF intervention has been further developed and modified to relate to a population of community-dwelling older adults, who are at risk of disability and therefore often in poorer physical health. Evidence of the motivators, barriers and challenges facing this population has been collected from a systematic synthesis of the qualitative literature on older adults and these data have led to the evolution and adaptation of the intervention for the PACE study. The PAF sessions are designed to be highly tailored to the individual participant’s circumstances, whilst staying true to the core principles of self-determination theory. The focus is on 'lifestyle' physical activity, that is including activity into an individual’s day-to-day life and daily routine, though participants interested in more formal exercise or sport sessions are also supported to achieve this.

### Measuring disability

In order to assess whether physical activity interventions make a clinically significant difference to the lives of older adults, it is necessary to identify an appropriate measure of disability. This is challenging because Nagi’s concept of disability requires a judgement of impairment in an individual’s social role which is difficult to assess in a research clinic setting. Previous studies have used a variety of outcomes including severe mobility limitation, nursing home admission, and self-reported measures of activities of daily living (ADL) [12]. However there are theoretical and practical limitations to these tools, particularly in randomised controlled trials (RCTs) where events such as nursing home admission may be rare, and self-report measures are susceptible to reporting bias, especially when participants are unblinded [13]. A measure of disability more recently used in research settings is ‘mobility disability’, that is the inability to complete a measured walking track within a specified time. Mobility disability is thought to represent a close approximation to disability, as it is assumed that the ability to mobilise is central to an older individual’s quality of life and ability to fulfil the activities of daily living [14].

Helpfully, a set of objective physical performance criteria has been identified that provides convincing prognostic information of the risk of age-related disability. The prognostic tool comprises measures of standing balance, gait speed, and chair rise and is known as the Short Physical Performance Battery (SPPB) [15]. Scores on the SPPB have been found to be highly predictive of new-onset disability, both self-reported and objective [16], and can predict loss of mobility, hospitalization, nursing home admission, and death. The SPPB measures may therefore be used as a screening tool to identify patients most likely to benefit from an intervention, and this approach has been used in several large-scale physical activity studies. In addition to their prognostic value, the SPPB, along with grip strength, also reflect an individual’s muscle strength and motor control. Since these functions are necessary for the basic tasks of independent living, the SPPB and grip strength are also appropriate outcome measures in studies looking at prevention of disability - they can be considered intermediary or proxy measures of disability.

### Hypotheses

It is hypothesised that the PAF intervention will be effective in increasing physical activity in older adults at risk of disability, and that the increased physical activity will lead to a reduction in disability and/or improved physical performance, as measured using a timed walk and the SPPB.

### Objectives

Before proceeding to a definitive RCT, it is important to ensure the feasibility of delivering both the intervention and a full-scale trial, as noted in the Medical Research Council (MRC) framework for the evaluation of complex interventions [17]. Therefore, an exploratory pilot trial is necessary to ensure that the PAF intervention may be delivered as intended; to ensure the intervention reaches the intended population; and to ensure that the recruitment and trial processes are successful and acceptable to participants.

The objectives of the exploratory pilot trial are:

1. To establish whether the PAF intervention is acceptable for the target population, and to collect data to test elements of the theoretical basis of the study (the programme theory)
2. To assess the feasibility of undertaking a definitive and full-scale RCT of the PAF intervention in the target population, evaluating the process of recruitment, screening, randomisation, collection of baseline and outcome data
3. To explore and describe any trial design aspects that may require modification before proceeding to a full-scale trial
4. To provide estimates of the variability around the important parameters, necessary to calculate the sample size and resources required for an adequately-powered full-scale trial.

As this study investigates a complex social intervention, the aim is to undertake a ‘realist RCT’, establishing what works, for whom, under what circumstances [18]. There will, therefore, be considerable emphasis on process evaluation and qualitative methodologies to understand underlying mechanisms.

## Methods

### Study design

The study is funded by the National Institute of Health Research (NIHR) as part of a Doctoral Research Fellowship award. Ethical approval was received from the Humber Bridge Research Ethics Committee (REC), later transferred to South Yorkshire REC (reference: 13/YH/0319).

The trial is a rater-blinded controlled trial using a concealed computer-generated random allocation sequence. Recruitment will run between April 2014 and January 2015. Outcomes are assessed at 6 months by an independent research nurse blinded to the allocation sequence. Qualitative interviews and observations will form part of a process evaluation to explore acceptability and assess intervention fidelity.

### Recruitment and randomisation

Sixty adults aged 65 years and over will be recruited from primary care practices across Bristol and the surrounding area. As this is a pilot study designed to explore feasibility, it will not be powered for a primary outcome. The sample size was selected to ensure sufficient numbers were available in both arms to provide useful data on process evaluation measures and variability in outcome measures. This sample size is consistent with other pilot studies of physical activity interventions [19,20]. The study will be open to both male and female participants; full eligibility criteria are listed in Table 1.

**Table 1:** **Eligibility criteria**

**Inclusion criteria**	
	Aged 65 years or older
	Community-dwelling, including those in sheltered accommodation
	Inactive: undertaking less than 150 minutes of moderate, or 75 minutes of vigorous, physical activity per week
	Non-disabled at baseline: able to complete a 4-metre walk at a speed of 0.8 m/s or greater, without sitting, leaning, using a walking aid or another person
	At risk of subsequent disability: scoring less than 10 out of 12 on the SPPB
**Exclusion criteria**	
	Unable to participate in the intervention or study due to speech, language, or sensory problems
	Resident in a nursing home
	Plans to move out of the area within 6 months of the screening clinic visit or to be away for more than 8 consecutive weeks during this period
	Currently participating in an exercise-on-prescription or rehabilitation programme or study
	A documented or patient-reported medical condition including but not limited to: severe arthritis; lung disease requiring home oxygen; serious cardiovascular disease; past history of cardiac arrest; implantable cardioverter defibrillator; neuromuscular or musculoskeletal conditions exacerbated by exercise; moderate or severe cognitive impairment or dementia; severe uncontrolled psychiatric illness; multiple falls
	Investigator concern about an individual’s safety or ability to adhere to the intervention

*Abbreviation*: *SPPB* Short Physical Performance Battery.

Up to six primary care practices will be selected from wards of differing deprivation level. We aim to evaluate alternative methods of recruitment in the study: conventional postal invitation and opportunistic recruitment by a researcher sitting in the waiting room. The effectiveness, acceptability, and feasibility of each recruitment method will be evaluated.

For practices using the postal invitation approach, general practitioners (GPs) will randomly select 375 patients from their list of eligible patients, excluding those they know to be ineligible, or those whom it would be inappropriate to approach (for example, those suffering from a recent bereavement). An invitation letter with a study information sheet will be sent to these patients. The invitation letter will contain a reply slip for interested individuals to make contact with the study team. Non-responders will be sent one reminder invitation. Interested individuals recruited through postal interviews will undergo telephone screening initially; this will enable exclusion of individuals who are already meeting the minimum recommended levels of physical activity, or who are unable to walk for a quarter of a mile. Patients eligible at this stage are invited to a clinical screening clinic.

In practices using opportunistic waiting room recruitment, older patients attending the practice for appointments will be approached by a researcher prior to their appointment. The researcher will check the basic eligibility criteria at this stage, and those interested and potentially eligible will be invited to a clinical screening appointment.

At the clinical screening appointment patients will be objectively assessed as ‘non-disabled’ using a timed 4-metre walk. This assessment is practical and highly predictive (>90%) of successfully completing the well-established 400-metre walk test within 15 minutes [21]. Individuals will also be assessed as ‘at risk of disability’ using the SPPB. Scores of less than 10 out of 12 on the SPPB represent up to a 5-fold increased risk of new-onset disability within 4 years [16]; this will be the threshold for inclusion in the PACE study and is consistent with the threshold used in other physical activity intervention trials [14].

Before the clinical screening appointment, potentially eligible patients will be sent a questionnaire in the post for prior completion, containing self-report measures of physical activity [22], motivation for physical activity, and mood [23]. At the clinical screening appointment patients will be asked about specific medical conditions that may mean physical activity is contraindicated, such as unstable angina or critical heart valve disease, based upon the eligibility criteria. Should the study investigator have concerns about the suitability of a patient for the study then these concerns may be discussed with the patient’s GP before enrolment.

Eligible patients will provide informed written consent and will be enrolled in the study immediately following the clinical screening appointment. Further baseline measures will be completed at this time (Table 2). Participants will also be provided with an ActiGraph GT1M accelerometer to be worn on the hip for 7 days and a journey log to document trips made during this period.

**Table 2:** **Baseline and follow-up measures**

	**Month 0**	**Month 0**	**Month 6**
Measure	Home	Baseline visit	Follow-up visit
**General**			
Medical and drug history		✓	
Sociodemographic details		✓	
Athropometry (weight ± height)		✓	✓
**Physical function and disability**			
Timed 4-m walk		✓	✓
Short Physical Performance Battery (SPPB)		✓	✓
Grip strength		✓	✓
Lawton’s scale of instrumental ADL		✓	✓
Physical activity			
ActiGraph GT1M accelerometer		✓	✓
PASE questionnaire	✓		✓
Mood			
Geriatric Depression Scale (15-part)	✓		✓
Cognitive			
Montreal Cognitive Assessment (MoCA)		✓	✓
Process measures			
Physical activity outcome expectations scale		✓	✓
Motivation for physical activity (BREQ-2) scale	✓		✓
Autonomy support scale (intervention group)			✓
Psychological need satisfaction in exercise^a^		✓	✓
Social Support Questionnaire (SSQ)		✓	✓
Basic Psychological Needs Scale		✓	✓
Quality of Life			
European Quality of Life 5 Dimensions (EQ-5D)		✓	✓
ICEpop CAPability measure for Older people		✓	✓
Health service use			
Primary and secondary care consultations			✓
Care home and hospital admission			✓

^a^NB in this scale the term 'exercise' will be replaced by 'physical activity'.

ADL, activities of daily living. BREQ, Behavioural Regulation in Exercise Questionnaire.

Randomisation will use a sequence minimising on age, gender, and GP practice. This will be computer-generated by the Bristol Randomised Trials Collaboration, a UK Clinical Research Collaboration (UKCRC)-registered clinical trials unit. The randomisation ratio will be 2:1 favouring the intervention arm (40 participants) over the control arm (20 participants). Once the participant has been allocated to either the intervention or control arm, this assignment will be made available to the researcher and participant: it is not possible to blind participants to the treatment allocation. Participants’ GPs are contacted following each enrolment and are briefed about the exclusion criteria, to ensure no patient is enrolled inappropriately.

### Treatment arms

Participants randomised to the intervention arm will be contacted by a trained PAF facilitator. It is anticipated that if cost-effective, the PAF intervention could be delivered by nurses or healthcare workers in community settings and so a background in physical activity or healthcare is a desirable, but not essential criteria for appointment. For the pilot trial 2 PAFs will be appointed and will receive a 3-day training course covering the theoretical background to the intervention, the principle behaviour change strategies relevant to the intervention, and an introduction to communication using motivational interviewing techniques. A comprehensive manual will be provided, and training sessions will be evaluated by observation, audio recording and transcription, and PAF feedback. PAF facilitators will have access to regular and frequent supervision and support throughout the trial.

Each intervention participant will receive an initial face-to-face PAF session, and after this participants will be offered up to two further face-to-face sessions, and up to nine telephone support sessions. These sessions are not at fixed intervals but are flexibly tailored to suit the participant and their progress. All intervention participants are offered the opportunity to involve a spouse, friend or close social partner in the sessions, or in supporting them to increase their activity levels. Worksheets designed to assist with behaviour change techniques such as goal setting and feedback are available for participants to use if they choose. PAF facilitators will keep detailed logs of each session and will reflect on how they are addressing the theoretical components of the intervention.

Participants allocated to the control arm will receive a booklet on healthy ageing, containing health promotion messages such as healthy diet, physical activity, and falls prevention. This is produced by Age UK, is publicly available and is, therefore, compatible with ‘usual care’.

### Measures and analysis

The main quantitative outcomes will be recruitment, adherence, and retention rates, in order to inform the design of a future full-scale trial. All participants will be followed-up 6 months after enrolment, and the baseline measures undertaken at month 0 will be repeated by a trained practice nurse or healthcare assistant who is blinded to the treatment allocation. Simple descriptive statistics will be used to describe the distribution and baseline variability of all outcomes of interest, and effect sizes may be calculated to assess for evidence of promise, whilst accepting that the study will not be powered to provide strong evidence against the null hypothesis. The variability of the parameters will contribute to a sample size calculation for the full-scale trial.

In the full-scale trial the primary outcomes will be the ability to complete the 4-metre walking test and the SPPB. Secondary outcomes will include physical activity (measured objectively and by self-report); body mass index (BMI); grip strength; and measures using the following tools: Lawton’s self-reported ADL [24]; Geriatric Depression Scale [23]; Montreal Cognitive Assessment (MoCA) [25]; and the Social Support Questionnaire (SSQ) [26]. Costs of the intervention will be documented, and healthcare use estimated from primary care records. The cost-effectiveness of the intervention may be estimated using data from the European Quality of Life 5 Dimensions (EQ-5D) [27] and ICECAP-O [28] quality of life tools. Full details of the measures collected in the pilot trial are shown in Table 2.

Adverse events may be expected in physical activity trials. Participants in both study arms will be asked about adverse events regularly; all adverse events will be reviewed and recorded. Serious adverse events will be reported to the sponsor and an independent clinical geriatrician, who will be responsible for discussing any concerns with the sponsor and advising the study team accordingly.

### Process evaluation

In order to evaluate whether the intervention operates as anticipated, process measures of physical activity expectations, motivations, and psychological needs satisfaction will be collected. These data will be supported by data gathered from qualitative interviews to assess whether the intervention is functioning at the levels expected. PAF sessions will be audio-recorded where possible and observed, allowing assessment of fidelity of the intervention. A purposefully-selected sample of participants will be asked to participate in a semi-structured interview. The interview will explore experiences of the intervention and trial, and attitudes and beliefs about physical activity. In addition, semi-structured interviews will be held with GPs, nurses, and primary care staff involved in the PACE study, and with the PAF facilitators, to explore their attitudes to the intervention and their experiences of the pilot trial. Interviews will be audio recorded, transcribed, and analysed using the Framework approach [29].

### Trial Advisory Committee

As this is a pilot study, no formal data monitoring committee will be convened. However the study has a Trial Advisory Committee (TAC), comprising an academic general practitioner, clinical geriatrician, and academics experienced in trial design, physical activity interventions in older adults, and statistics. The TAC provides advice on study design, adverse events, and data analysis.

## Discussion

This pilot RCT and exploratory study aims to assess the feasibility of using a novel theory-based intervention, customised for an older population, as a means of increasing physical activity and physical performance. When evaluating a new individual-level intervention, first targeting those with the greatest capacity to benefit often leads to the most efficient use of limited resources. It is thus important to focus initially on identifying a healthy population who are ‘at risk’ of future disability. Should the intervention appear effective in this group, it may then be appropriate to evaluate the intervention whilst adopting a ‘population approach’, accepting that the individual health gain to individuals at low risk is unlikely to be as great as to those at higher risk of disability. Ultimately however, if the intervention is shown to be cost-effective, eventual implementation of the PAF intervention in routine care could impart considerable public health benefits, and lead to substantial improvements in quality of life for older adults.

### Trial status

Recruiting.

## Abbreviations

ADL: activities of daily living
BMI: body mass index
GP: general practitioner
ICECAP-O: ICEpop CAPability measure for Older people
MoCA: Montreal Cognitive Assessment
MRC: Medical Research Council
PACE: Physical ACtivity for Elders
PAF: Physical Activity Facilitation
RCT: randomised controlled trial
REC: Research Ethics Committee
SPPB: Short Physical Performance Battery
SSQ: Social Support Questionnaire
TAC: Trial Advisory Committee
TREAD: TReatment with Exercise Augmentation for Depression
UK: United Kingdom
UKCRC: UK Clinical Research Collaboration
